# The Downregulation of PTGS2 Mediated by ncRNAs is Tightly Correlated with Systemic Sclerosis-Interstitial Lung Disease

**DOI:** 10.3389/fgene.2021.795034

**Published:** 2022-01-13

**Authors:** Zhixiao Xu, Chengshui Chen

**Affiliations:** ^1^ Department of Pulmonary and Critical Care Medicine, The First Affiliated Hospital of Wenzhou Medical University, Wenzhou, China; ^2^ The Interventional Pulmonary Key Laboratory of Zhejiang Province, Wenzhou, China

**Keywords:** PTGS2, systemic sclerosis-interstitial lung disease, immunocyte infiltration, tumor growth, non-coding RNA

## Abstract

**Background:** Interstitial lung disease in systemic sclerosis (SSc-ILD) is one of the most severe complications of systemic sclerosis (SSc) and is the main cause of mortality. In this study, we aimed to explore the key genes in SSc-ILD and analyze the relationship between key genes and immune cell infiltration as well as the key genes relevant to the hallmarks of cancer.

**Methods:** Weighted gene co-expression network analysis (WGCNA) algorithm was implemented to explore hub genes in SSc-ILD samples from the Gene Expression Omnibus (GEO) database. Logistic regression analysis was performed to screen and verify the key gene related to SSc-ILD. CIBERSORT algorithms were utilized to analyze immune cell infiltration. Moreover, the correlation between the key genes and genes relevant to cancer was also evaluated. Furthermore, non-coding RNAs (ncRNAs) linking to PTGS2 were also explored.

**Results:** In this study, we first performed WGCNA analysis for three GEO databases to find the potential hub genes in SSc-ILD. Subsequently, we determined PTGS2 was the key gene in SSC-ILD. Furthermore, in CIBERSORT analyses, PTGS2 were tightly correlated with immune cells such as regulatory T cells (Tregs) and was negatively correlated with CD20 expression. Moreover, PTGS2 was associated with tumor growth. Then, MALAT1, NEAT1, NORAD, XIST identified might be the most potential upstream lncRNAs, and LIMS1 and RANBP2 might be the two most potential upstream circRNAs.

**Conclusion:** Collectively, our findings elucidated that ncRNAs-mediated downregulation of PTGS2, as a key gene in SSc-ILD, was positively related to the occurrence of SSc-ILD and abnormal immunocyte infiltration. It could be a promising factor for SSc-ILD progression to malignancy.

## Introduction

Systemic sclerosis (SSc) is an auto-immune “orphan” disease, which is often accompanied by progressive fibrosis of some internal organs, most common of which is lung. Pulmonary complications should be given more attention as they are the leading cause of mortality in SSc patients (Denton and Khanna, 2017). Interstitial lung disease in systemic sclerosis (SSc-ILD) and idiopathic pulmonary fibrosis (IPF), both of which target the lung, are distinct lung diseases (Herzog et al., 2014). Although dysregulated fibroblast activation and myofibroblast accumulation are present in both conditions, there are substantial differences in the initiating events and pathways of disease perpetuation and progression (Mattoo and Pillai, 2021). Persistent alveolar and airway epithelial injury defines the core of IPF pathogenesis, while SSc-ILD exhibits defects in endothelial cell homeostasis (Solomon et al., 2013). Therefore, there is still much more knowledge about the biological bases for disease presentation which could affect clinical diagnosis and patient management.

There are a growing number of genomic approaches to diagnosis and prognostication of SSc-ILD, including identification of candidate genes, such as CD226, CD247, CTGF, IRAK1, IRF5, NLRP1, and STAT4 (Stock and Renzoni, 2018). As a consequence, both a better understanding of the underlying mechanisms involved in SSc-ILD and the discovery of novel biomarkers for this type of disease are major clinical challenges.

The aim of our project was to explore the key genes in SSc-ILD and analyze the relationship between key genes and immune cell infiltration as well as the key genes relevant to the hallmarks of cancer.

## Materials and Methods

### Co-Expression Network Analysis

Data acquisition from public database. Gene matrix expression profiles of SSc-ILD were obtained from Gene Expression Omnibus (GEO) (GSE48149, GSE76808, and GSE81292). All genes in each dataset were sorted by logFC and normalized using the “normalizeBetweenArrays” function.

In order to identify key modules, we processed gene matrix expression profiles and clinical values through the weighted correlation network analysis (WGCNA). Later, we applied the means method to implement hierarchical clustering, and only considered 18 subjects in GSE76808 in which genes with the coefficient of variation (CV) > 7% were extracted. Furthermore, an analysis of network topology was used to determine a soft-thresholding power less than 12. In a subsequent analysis, we merged modules with high similarity and identified a new set of modules that were automatically assigned indicating their size color by WGCNA.

### Screening of the Hub Genes

Genes with intramodular connectivity values of >0.85 and gene importance >0.85 in the module most relevant to SSc-ILD were extracted for subsequent analysis. Additional datasets GSE48149 and GSE81292 were used to evaluate the filtering genes. Finally, the hub genes that were consistently up-regulated or down-regulated in the three cohorts were screened.

To further evaluate the diagnostic value of hub genes in the diagnosis of SSc-ILD, univariate logistic regression analysis and receiver operating characteristic (ROC) curve analysis were performed based on three GEO databases.

### Relationship Between PTGS2 Expression Level and Immune Cell Infiltration as Well as Immunomodulators in Systemic Sclerosis-Interstitial Lung Disease

Through the “IORB” package (Zeng et al., 2021), CIBERSORT algorithm was used to analyze the correlation of the expression level of PTGS2 with immune cell infiltration levels. Furthermore, immunomodulators were downloaded from TISIDB (http://cis.hku.hk/TISIDB/), to explore the correlation between PTGS2 expression level and the expression levels of immunomodulators in SSc-ILD. Later, the expression of immunomodulators between the two groups divided by the median value of PTGS2 expression level was evaluated.

### Relationship Between PTGS2 and Key Genes Relevant to Cancer

The close relationship between SSc-ILD and cancer was considered. GSE48149, GSE76808, and GSE81292 databases were used to evaluate the correlation between PTGS2 expression level and the expression levels of key genes relevant to the hallmarks of cancer including proliferation, invasion, epithelial-mesenchymal transition, angiogenesis, and lymph-angiogenesis.

### Candidate miRNA and lncRNA Prediction and Evaluation

Upstream binding miRNAs of the key gene were predicted through starBase (http://starbase.sysu.edu.cn/). Only the predicted miRNAs that commonly appeared in more than four programs (containing PITA, RNA22, miRmap, microT, miRanda, PicTar, and TargetScan) were included for subsequent analyses. In addition, starBase was used to predict candidate lncRNAs and cirRNAs that could potentially bind to the miRNA selected.

## Results

### Key Genes Were Identified in Systemic Sclerosis-Interstitial Lung Disease

**FIGURE 1:**
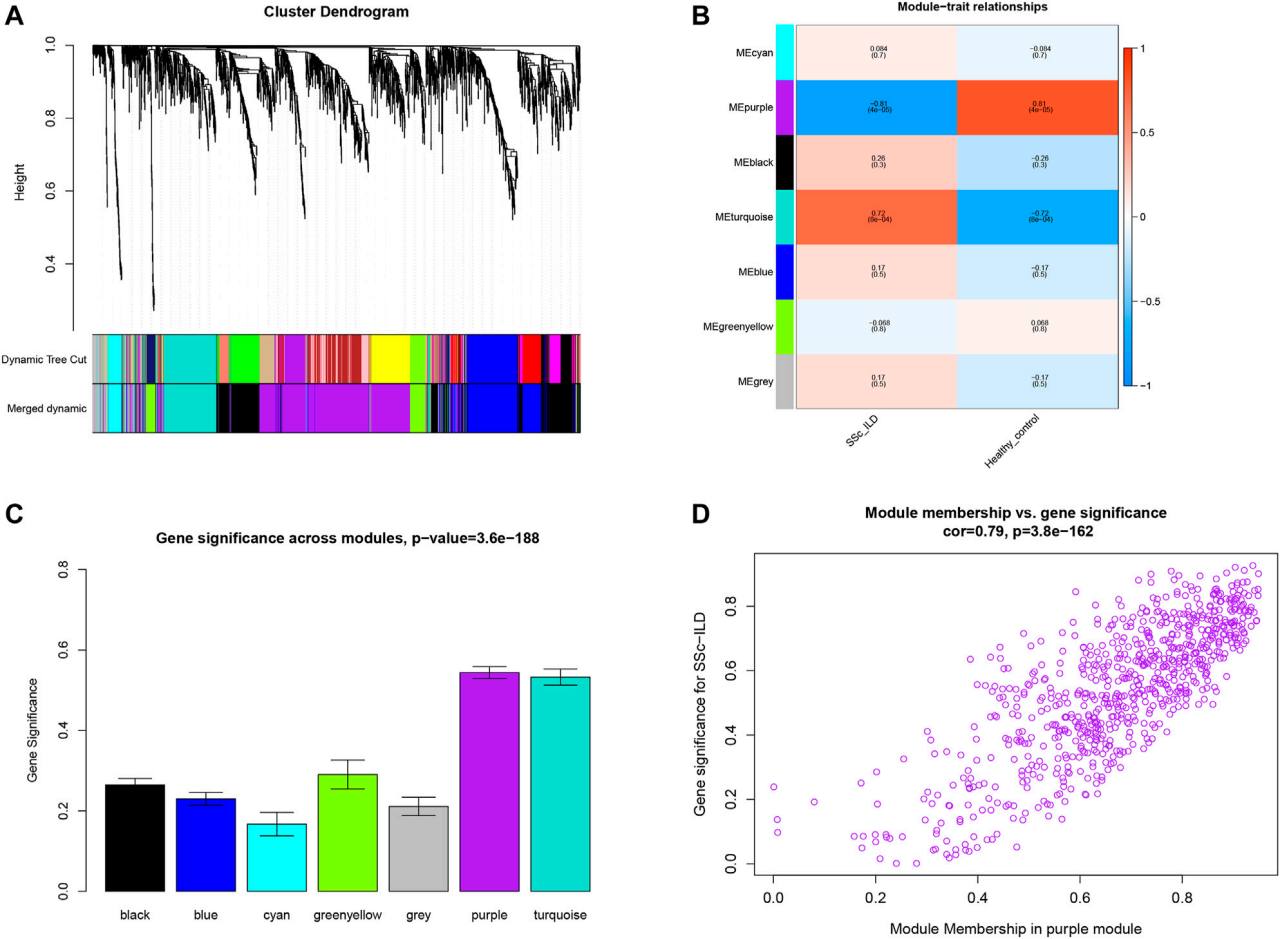
**(A)** Clustering dendrogram of genes which coefficient of variation >7% associated with systemic sclerosis-related interstitial lung disease (SSc-ILD) in GSE76808. **(B)** Heatmap of module-trait relationships revealed by WGCNA analysis. **(C)** Distribution of average gene significance and errors in the modules related to SSc-ILD. **(D)** The gene significance for SSc-ILD in the purple module (one dot represents one gene in the purple module).

WGCNA was executed to construct a gene co-expression network to identify a highly synergistic gene set based on weighted gene expression correlation. Seven co-expression modules were constructed by WGCNA analysis (Figure 1A). The purple gene module was ascertained to have the most negative correlation with SSc-ILD (Cor = −0.81, *p* < 0.05) (Figure 1B-D). Based on the cut-off criteria, 130 genes with high connectivity in the clinically important module were identified as hub genes. It was worth noting that ten genes were downregulated in the three cohorts consistently (Table 1). It indicated that the ten genes were tightly correlated with SSc-ILD.

**TABLE 1:** The common genes identified in the microarray dataset GSE48149, GSE76808, and GSE81292.

Genesymbol	GSE48149		GSE76808		GSE81292	
	logFC	adj.P.Val	logFC	adj.P.Val	logFC	adj.P.Val
ABL2	−0.52948	0.04264	−0.70701	0.002468	−1.092	0.000514
IL6	−1.60993	0.021782	−4.23683	6.97E-05	−4.63817	3.15E-06
IRF1	−1.04897	0.024041	−1.41216	7.49E−05	−1.13	1.11E−05
LIF	−0.9891	0.017001	−2.09567	3.49E−05	−1.88167	0.001086
LMNA	−0.83824	1.98E−05	−1.40368	0.00037	−0.991	0.000128
MMP19	−1.1232	0.008257	−2.03139	0.00043	−1.37567	2.28E−05
MT1F	−0.7114	0.014012	−1.5723	0.000308	−0.9175	0.000783
PELI1	−0.73432	0.008873	−1.26774	3.35E−05	−1.332	1.11E−05
PTGS2	−1.29439	0.008257	−2.05182	0.00124	−2.13617	0.000135
TIPARP	−0.69919	0.018225	−1.4468	0.000683	−1.0915	0.001232

### PTGS2 Played an Important Role in Systemic Sclerosis-Interstitial Lung Disease

**FIGURE 2:**
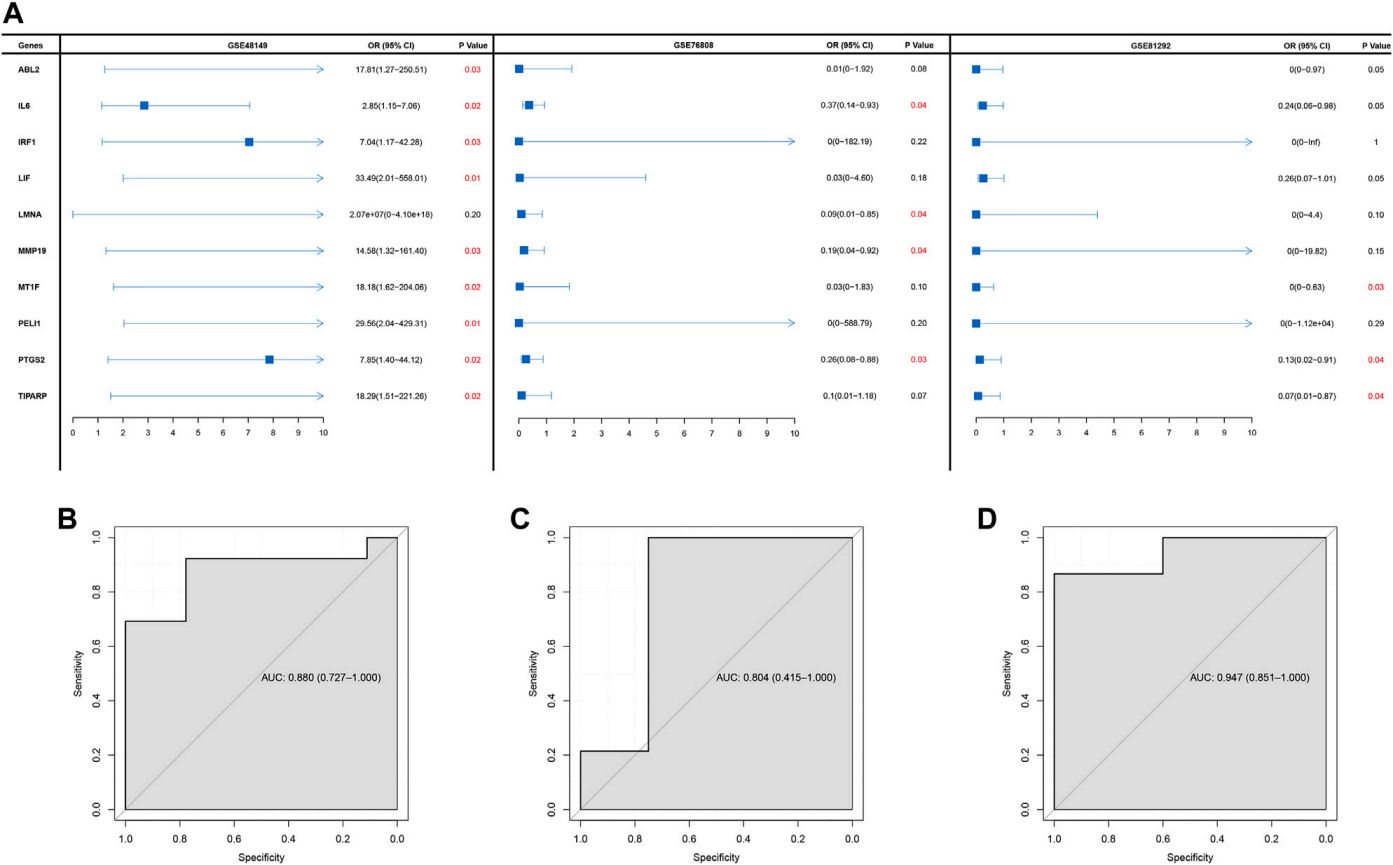
**(A)** Forest plot of logistic analysis in GSE48149, GSE76808, and GSE81292 datasets. **(B–D)** ROC curves in GSE48149 (B), GSE76808 (C), and GSE81292 (D) datasets.

Univariate logistic regression was executed to ascertain the diagnostic value of hub genes in SSc-ILD based on the three GEO cohorts. Finally, only one gene PTGS2 that was significantly related to SSc-ILD was identified (Figure 2A). Moreover, the AUCs were 0.880 (95%CI: 0.727–1.000), 0.804 (95%CI:0.415–1.000), 0.947 (0.851–1.000), respectively, in GSE48149, GSE76808, and GSE81292. The ROC curves analysis indicated that the gene PTGS2 had high sensitivity and specificity in the diagnosis of SSc-ILD (Figures 2B–D).

### PTGS2 Played a Critical Role in the Immune System

**FIGURE 3:**
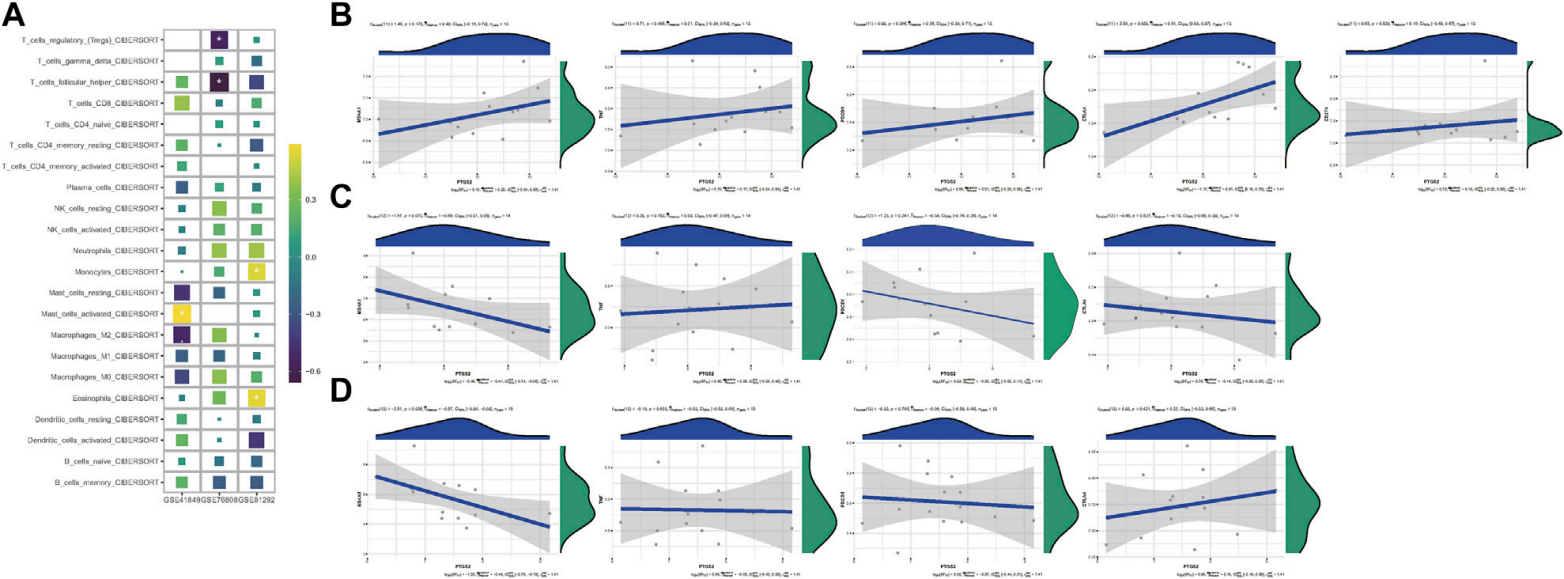
**(A)** Heatmap depicting the relationship between the CIBERSORT scores of 22 immune cells and the PTGS2 expression in GSE48149, GSE76808, and GSE81292 datasets. *p*-values were showed as: ns, not significant; **, *p* < 0.05; **, *p* < 0.01; ***, *p* < 0.001. **(B)** Spearman correlation of PTGS2 expression level with the expression of MS4A1 (CD20), TNF (TNFα), PDCD1, CTLA4, and CD274 (PD-L1) in GSE48149. **(C)** Spearman correlation of PTGS2 expression level with the expression of MS4A1 (CD20), TNF (TNFα), PDCD1, and CTLA4 in GSE76808. **(D)** Spearman correlation of PTGS2 expression level with the expression of MS4A1 (CD20), TNF (TNFα), PDCD1, and CTLA4 in GSE81292.

In CIBERSORT analyses, eosinophils, activated mast cells, monocytes, follicular helper T cells, and regulatory T cells (Tregs) were tightly related with PTGS2 expression level (Figure 3A). The relationships between PTGS2 expression level and the expression of MS4A1(CD20), TNF (TNFα), PDCD1, CTLA4, and CD274 (PD-L1) were also evaluated (Figure 3B–D). PTGS2 expression level was positively correlated with CTLA4 (PD-L1) expression level in GSE48149 (Figure 3B), while PTGS2 expression level was negatively correlated with MS4A1 (CD20) expression level in GSE81292 (Figure 3D). The expression of immunomodulators between the two groups divided by the median value of PTGS2 expression level was evaluated. The expression of immunomodulators between the two groups divided by the median value of PTGS2 expression level indicated that PTGS2 was related to immunomodulators (Supplementary Figure S1).

### PTGS2 Was Associated With Tumor Growth

**FIGURE 4:**
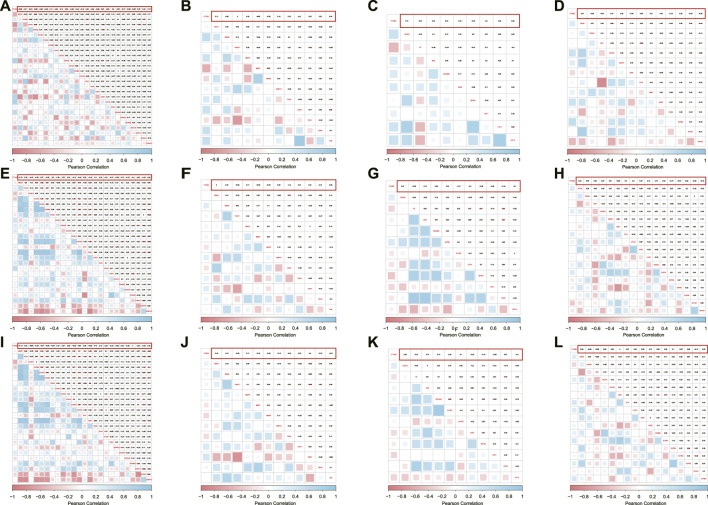
Correlation between PTGS2 expression and cell proliferation/cell cycle regulators were shown as heatmap in GSE48149 **(A)**, GSE76808 **(E)**, and GSE81292 **(I)**; Correlation between PTGS2 expression and MMPs/EMT and stemness marks were shown as heatmap in GSE48149 **(B)**, GSE76808 **(F)**, and GSE81292 **(J)**; Correlation between PTGS2 expression and angiogenesis markers were shown as heatmap in GSE48149 **(C)**, GSE76808 **(G)**, and GSE81292 **(K)**; Correlation between PTGS2 expression and lymph-angiogenesis markers were shown as heatmap in GSE48149 **(D)**, GSE76808 **(H)**, and GSE81292 **(L)**.

To explore the molecular machinery underlying the implication of PTGS2 in the progression of SSc-ILD disease into tumors, correlation between PTGS2 and key genes relevant to the hallmarks of cancer was determined. PTGS2 exhibited significant inverse correlation with MKI67 in GSE48149, but no correlation in the other two datasets (Figures 4A,E,I). Moreover, certain PTGS2 was inverse correlated with cell cycle promoting factors such as CCNA1, CCNA2, and CDK3, while a positive correlation was evident for cell cycle inhibitors such as CCND2, CDK4, CDK6 and CDK7. PTGS2 had not tightly relationship with the regulators of invasion/migration, angiogenesis, and lymph-angiogenesis/lymph node metastasis in cancer (Figure 4).

### Prediction and Analysis of Upstream miRNA, lncRNA and circRNAs of PTGS2

**FIGURE 5:**
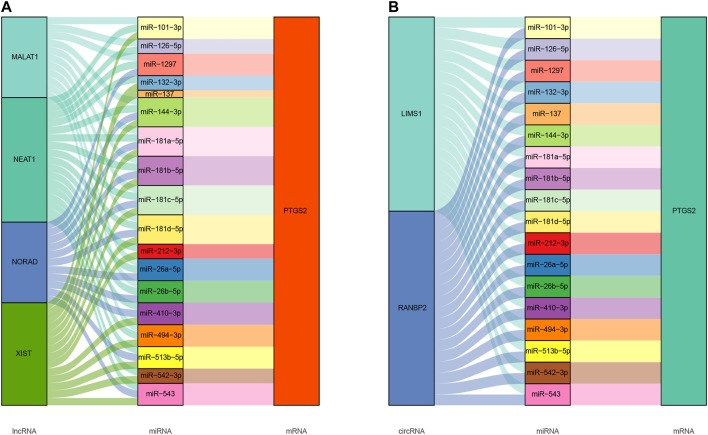
**(A)** The lncRNA-miRNA-mRNA ceRNA Sankey plot was constructed via 4 lncRNAs, 18 miRNAs, and PTGS2 for SSc-ILD; **(B)** The circRNA-miRNA-mRNA ceRNA Sankey plot was constructed via 2 circRNAs, 18 miRNAs, and PTGS2 for SSc-ILD.

ncRNAs are responsible for the regulation of gene expression, which has been widely recognized. To ascertain whether PTGS2 was modulated by some ncRNAs, upstream miRNAs that could potentially bind to PTGS2 were predicted and finally 18 miRNAs were found. Next, the upstream lncRNAs of 18 miRNAs were predicted through starBase database, there were 4 lncRNAs were tightly correlated with 18 miRNA, and 2 circRNAs were tightly correlated with 18 miRNAs. Visually, the ceRNAs network was established (Figure 5A,B). Taking correlation analysis into consideration, MALAT1, NEAT1, NORAD, XIST might be the most potential upstream lncRNAs, and LIMS1 and RANBP2 might be the two most potential upstream circRNAs.

## Discussion

The interplay among inflammation, autoimmunity and fibrosis seems to play an indispensable role in SSc (Scala et al., 2004). SSc exhibits a great deal of heterogeneity, probably due to a complex network of interactions between structural and inflammatory components, including different cell types, cytokines and chemokines, and components of the extracellular matrix (Mattoo and Pillai, 2021). Moreover, metabolites and enzymes of the arachidonic acid cascade, including the Cox-2 enzyme, are considered to be critical mediators of the inflammatory response (Allanore et al., 2008). In addition, Cox-2 also was called PTGS2.

The negative association of PTGS2 expression and SSc-ILD, found in the present study, has been supported by some research. Serum PTGS2 levels were diminished in SSc-ILD patients (Bassyouni et al., 2012). Furthermore, in IPF, PTGS2 loss occurred in bronchial epithelial cells, but not macrophages (Petkova et al., 2003). In animal models, PTGS2 deficient mice promoted bleomycin-induced pulmonary fibrosis (Keerthisingam et al., 2001). Lovgren et al. have reported that COX-2-derived prostacyclin protected both the development of fibrosis and the consequential alterations in lung mechanics (Lovgren et al., 2006). Similarly, cultured lung fibroblasts isolated from IPF patients have an impaired ability to synthesize prostaglandin E2 (PGE2) and to express PTGS2 (Wilborn et al., 1995).

Results from studies conducted in recent years suggest that PTGS2 and its metabolites mediate a protective effect on pulmonary fibrosis. A growing body of evidence implies that the feedback relationship between matrix sclerosis, PTGS2 inhibition, and fibroblast activation could promote and amplify progressive fibrosis. In the presence of elevated levels of pro-fibrotic factors, this decreased ability to upregulate PTGS2 expression may lead to unimpeded fibroblast proliferation and collagen synthesis, thereby contributing to the pathogenesis of IPF (Matsumura et al., 2009; Stratton and Shiwen, 2010). Although tissue sclerosis has traditionally been regarded as a consequence of fibrosis rather than a contributing factor to pathogenesis, Liu et al. have shown that increased matrix stiffness strongly inhibits fibroblast expression of PTGS2 synthesis and that exogenous PGE2 completely counteracts the proliferative and matrix synthesis effects induced by this increased stiffness (Liu et al., 2010). Epigenetic abnormalities in a subset of key genes involved in tissue remodeling, as observed in IPF, result in reduced transcription of PTGS2 genes (Coward et al., 2009; Coward et al., 2010; Pang and Zhuang, 2010).

Over the past decade, many biologics have been tested in both IPF and SSc, but the results in the clinical settings have been mostly unsatisfactory. For SSc, immunosuppressive treatments such as cyclophosphamide (CYC) and mycophenolate mofetil (MMF) remain the main therapeutic option, especially in SSc-ILD despite its caveats (Tashkin et al., 2016; Kowal-Bielecka et al., 2017; Volkmann et al., 2019). However, one study showed a significant and persistent benefit for patients treated with Rituximab (anti-CD20) on top of standard treatment compared to standard treatment alone (Daoussis et al., 2017). Given the high level of auto-antibodies and the presence of B cell infiltrates in skin samples of SSc patients, Rituximab (anti-CD20), which depletes B cells, has been recently tested with some success, especially in CYC refractory patients (Fernández-Codina et al., 2018; Elhai et al., 2019). In our study, PTGS2 was negatively correlated with MS4A1 (CD20) in GSE81292, suggesting that CD20 played a role in SSc-ILD possibly through the PTGS2-associated pathways.

Several population-based SSc cohort studies have reported an increased incidence of malignancy (Hill et al., 2003; Chatterjee et al., 2005; Olesen et al., 2010; Onishi et al., 2013). The tumor-infiltrating regulatory T cells (Treg) counts were positively correlated with intra-tumoral PTGS2 expression, particularly in patients with lymph node-negative NSCLC (Shimizu et al., 2010). The CD8-positive tumor-infiltrating lymphocytes counts were inversely correlated with intra-tumoral PTGS2 expression in patients with lymph node-negative LUAD (Shimizu et al., 2017). In our CIBERSORT analyses, eosinophils, activated mast cells, monocytes, follicular helper T cells, and regulatory T cells (Tregs) were tightly related with PTGS2 in SSc-ILD, which reflected the tight relationship between SSc-ILD and malignancy.

Researchers have long noticed the important role of PTGS2 in the occurrence and development of cancer. In inflammation-associated carcinogenesis, PTGS2 is markedly overexpressed, leading to accumulation of various prostaglandins with oncogenic potential (Choi et al., 2020). Inflammation is a key mediator of angiogenesis and lymph-angiogenesis with aberrant expression of PTGS2 (Linares et al., 2021), and aberrant expression of PTGS2 was associated with tumor growth in the present study, but it has not been clearly elucidated and further functional biological experiments are required.

Moreover, the ceRNA constructed was composed of 18 miRNA nodes, 4 lncRNA nodes, and 2 circRNA nodes. A study reported that downregulation of miR-542-3p contributed to apoptosis resistance in dermal fibroblasts from SSc patients through survivin overexpression (Manesh et al., 2019).

However, there are several limitations in our study. First, this study was conducted only based on the GEO database. Given the low prevalence of SSc-ILD and the fact that not all SSc-ILD patients underwent lung biopsy, we integrated three different microarray datasets of SSc-ILD, but the sample size was still limited. Secondly, the analysis of immune cell infiltration was based on the CIBERSORT algorithm and further studies are needed to investigate the complete picture of infiltrating immune cells in SSc-ILD as the heterogeneity and complexity of the immune microenvironment was not taken into account. Thirdly, the potential target miRNA and lncRNAs were predicted using the online tool, which needs experimental verification.

## Conclusion

Our study uncovered that decreased PTGS2 was positively related to occurrence of SSc-ILD, and abnormal immunocyte infiltration. It could be a promising factor in the development of SSc-ILD into malignant tumor. And MALAT1, NEAT1, NORAD, XIST might be the most potential upstream lncRNAs, and LIMS1 and RANBP2 might be the two most potential upstream circRNAs.

## Data Availability

The original contributions presented in the study are included in the article/Supplementary Material, further inquiries can be directed to the corresponding author.
