# Supplementary figures and images for: The Downregulation of PTGS2 Mediated by ncRNAs is Tightly Correlated with Systemic Sclerosis-Interstitial Lung Disease
Source: Front Genet. 2022 Jan 13;12:795034. doi: 10.3389/fgene.2021.795034 (PMC8793859; doi:10.3389/fgene.2021.795034)

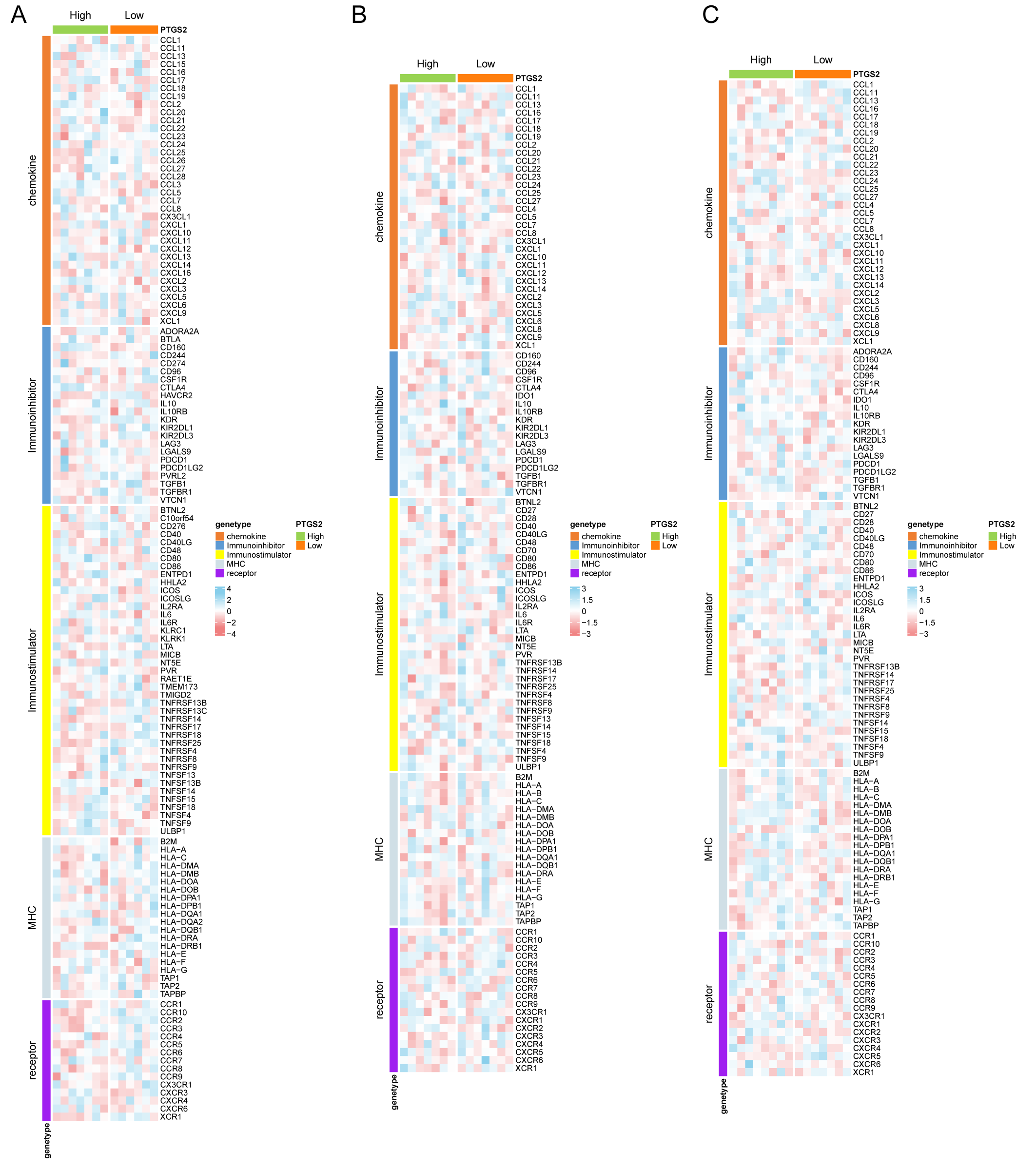

Supplement: Supplementary file 1 [file Image1.TIF]
